# Evolutionary adaptation to climate change

**DOI:** 10.1093/evlett/qrad070

**Published:** 2024-02-13

**Authors:** Allan H Edelsparre, Mark J Fitzpatrick, Marjo Saastamoinen, Céline Teplitsky

**Affiliations:** Department of Ecology and Evolutionary Biology, University of Toronto, Toronto, ON, Canada; Department of Biological Sciences, University of Toronto Scarborough, Toronto, ON, Canada; Faculty of Biological and Environmental Sciences, University of Helsinki, Helsinki, Finland; Institute of Life Sciences, University of Helsinki, Helsinki, Finland; CEFE, Univ Montpellier, CNRS, EPHE, IRD, Montpellier, France

**Keywords:** adaptation, epigenetics, climate change, evolutionary genomics, prediction

## Abstract

When the notion of climate change emerged over 200 years ago, few speculated as to the impact of rising atmospheric temperatures on biological life. Tens of decades later, research clearly demonstrates that the impact of climate change on life on Earth is enormous, ongoing, and with foreseen effects lasting well into the next century. Responses to climate change have been widely documented. However, the breadth of phenotypic traits involved with evolutionary adaptation to climate change remains unclear. In addition, it is difficult to identify the genetic and/or epigenetic bases of phenotypes adaptive to climate change, in part because it often is not clear whether this change is plastic, genetic, or some combination of the two. Adaptive responses to climate-driven selection also interact with other processes driving genetic changes in general, including demography as well as selection driven by other factors. In this Special Issue, we explore the factors that will impact the overall outcome of climate change adaptation. Our contributions explain that traits involved in climate change adaptation include not only classic phenomena, such as range shifts and environmentally dependent sex determination, but also often overlooked phenomena such as social and sexual conflicts and the expression of stress hormones. We learn how climate-driven selection can be mediated via both natural and sexual selection, effectively influencing key fitness-related traits such as offspring growth and fertility as well as evolutionary potential. Finally, we explore the limits and opportunities for predicting adaptive responses to climate change. This contribution forms the basis of 10 actions that we believe will improve predictions of when and how organisms may adapt genetically to climate change. We anticipate that this Special Issue will inform novel investigations into how the effects of climate change unfold from phenotypes to genotypes, particularly as methodologies increasingly allow researchers to study selection in field experiments.

## Introduction

When the notion of global warming first emerged over 200 years ago (Arrhenius, 1896), few thought that human activity was capable of altering the global climate. Consequently, the urge to understand how changes in global temperatures could affect Earth’s ecosystems and biodiversity was limited. Fast forward to the present day, and we have arrived at a reality where nearly all ecological processes, including ecosystem function and services, are affected by a globally changing climate induced by human activities (Scheffers et al., 2016). Thus, the accelerating effects of climate change on biodiversity remain the nexus of our current climate crisis as more than a million species now directly face the risk of extinction (Díaz et al. 2019; IPBES, 2019), although extinctions only represent the tip of the iceberg (Fraixedas et al., 2022).

Historically, the successful tackling of environmental crises caused by human societies has been fostered by a deep understanding of the biological processes involved. For example, major environmental crises caused by man-made chemicals, such as ozone-depleting substances and DDT, were unraveled and tackled by understanding how the chemicals entered and interfered with entire food chains, ultimately causing cancer and genetic damage to organisms. The current climate crisis differs from historical ones in that ecosystem change is on a global scale wherein the effects on global biodiversity are both highly unpredictable and continually advancing (Marquet et al., 2019). Even if we successfully decreased the warming of the atmosphere, we find ourselves with limited alternatives but to allow the climate warming scenario forecasted for the 21st century to unfold (IPCC, 2022; Marquet et al., 2019; Nadeau et al., 2017). These alterations in climatic conditions will lead many species to adapt or face extinction. Thus, climate change has become an unfortunate global experiment with evolutionary adaptation at its core, and the study of evolutionary rescue plays a major role in predicting which species manage to persist, consequently influencing the future of all ecosystems.

**Figure 1.**
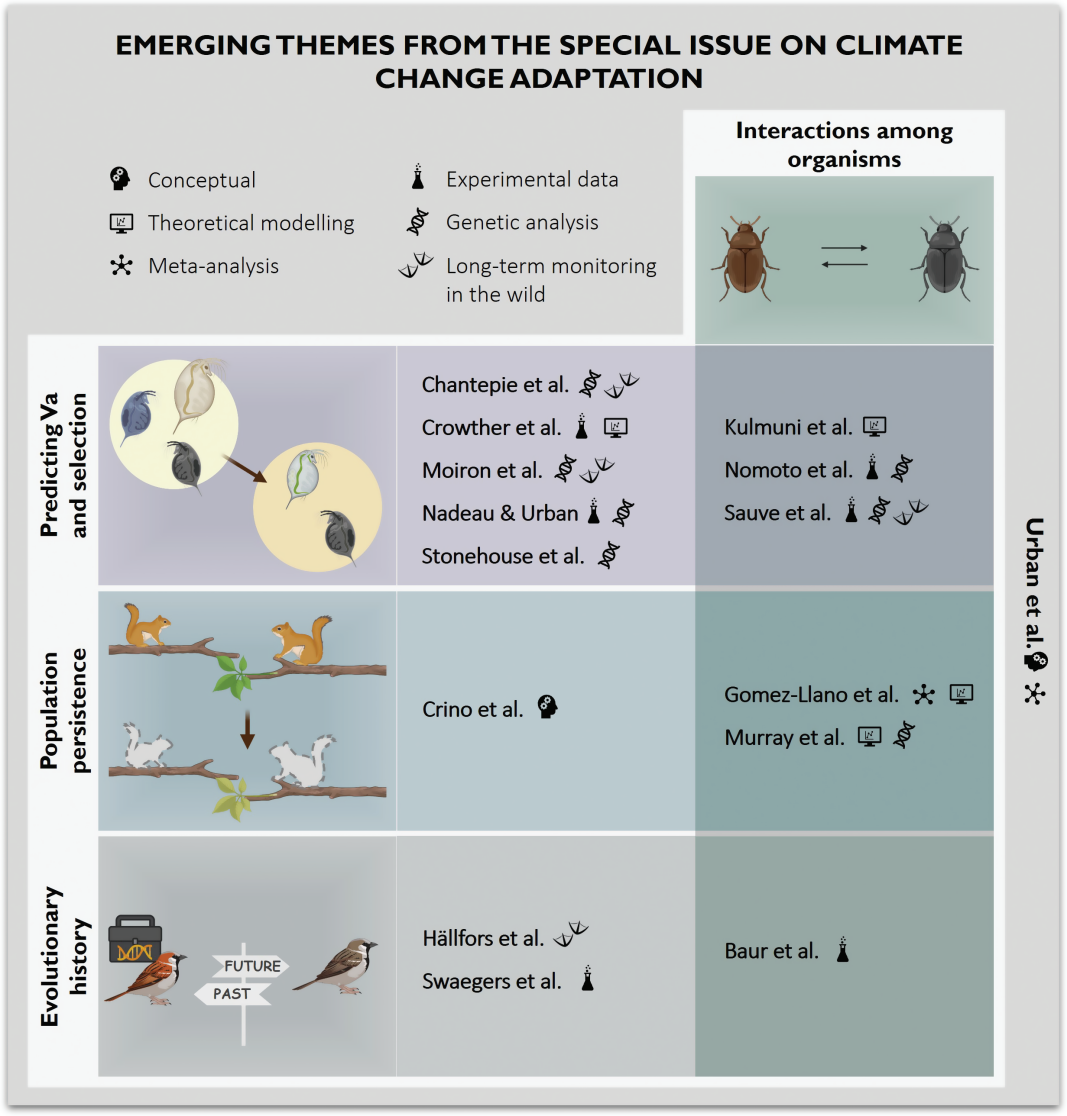
Schematic illustration of the key themes that emerged from the 15 contributions in the Special Issue: Evolutionary Adaptation to Climate Change, such as predicting Va (e.g., additive genetic variance), population persistence, and evolutionary history. Interactions among organisms include both within- and among-species interactions. Several contributions could fit in more than two of these themes, but here we highlight only the main aspects. Experiments ranged from short-term reaction norm experiments to experimental evolution studies. Genetic analyses cover both genomic and quantitative genetic studies, and long-term monitoring in the wild refers to species occurrence and individual monitoring data.

There are two key questions. First, how will species adapt to climate change. Second, which critical factors best predict which species (or populations) can adapt and be rescued by evolution in the face of a rapidly changing climate? An important part of answering these questions lies rooted within the relevant traits that are directly or indirectly influenced by climate change (Urban et al., 2024, 172–187). Many reports have already demonstrated that climate change has influenced the distribution patterns of many species and altered phenomena such as migration, the timing of events such as reproduction (Inouye, 2022; Walther et al., 2002), or diapause (Bradshaw & Holzapfel, 2001). However, it remains unknown why such responses are evident in some, but not all, species influenced by climate change. In cases where responses to climate change are evident, studies rarely demonstrate whether such changes are caused by plastic responses, genetic changes, or some combination of the two (Bonnet et al., 2019; Merilä & Hendry, 2014; Ramakers et al., 2019). In fact, studies rarely investigate whether selection in the wild is driven by climate change (Bonnet et al., 2019). In addition, adaptive responses to climate change-driven selection may interact with other ecological and evolutionary processes that drive genetic changes in general (e.g., dispersal, demography, species interactions, and sexual selection). These factors represent major limitations to our understanding of “whether” and “how” species will adapt to climate change. Moreover, without uncovering how such interactions unfold it will be difficult to predict evolutionary outcomes promoted by climate change (e.g., Pelletier et al., 2009; Pujol et al., 2018). Accordingly, a section of this Special Issue is dedicated to studies that investigate traits that respond directly to selection, including the role of traits in mediating evolutionary adaptive responses to climate change and the plastic/genetic bases of these responses. A second section is dedicated to studies that investigate the impact of climate change on selection and evolutionary potential. The contributions in this section shed light on the complex relationship between climate-driven evolutionary change and change driven by ecological processes in general. In a concluding article, we merge the perspectives of many of the contributors of this Special Issue to develop a road map for predicting adaptive responses to climate change. We do this by exploring what we may be able to currently predict, opportunities that are likely to advance future predictions, and factors that we likely will not be able to predict (or may not even need to predict). In the current article, we highlight some transversal themes that emerged from the Special Issue (Figure 1).

## Predicting phenotypic responses to climate change

Predicting adaptive responses is a long-standing challenge. Historically, predicting adaptive evolution has been the aim of quantitative genetics (Roff, 2007), and is notoriously challenging in the wild (Kruuk et al., 2008; Pujol et al., 2018). Therefore, comparisons between predicted and estimated responses to selection in the wild in the context of climate change are scarce (but see e.g. Gienapp et al., 2006; Moiron et al., 2024, 8–17). Data sets from long-term monitoring are ideal for bridging such gaps. Using a long-term pedigree data set, Moiron et al. (2024, 8–17) show that arrival dates in migrating common terns (*Sterna hirundo*) are shifting earlier in the season. Theoretical models predict earlier arrival, and accordingly, they do find ongoing evolution in the population. However, according to Moiron et al. (2024, 8–17), both empirical and predicted genetic trends fall short compared with actual arrival date trends, suggesting that a part of the response involves plasticity. Genomic approaches offer the possibility of exploring adaptation beyond single traits, for example over a wide range of unmeasured traits. Using Gene Ontology, Stonehouse et al. (2024, 18–28) identify genomic regions in 20 populations of great tits (*Parus major*) across the entire European range that have responded to past and present climates. In an elegant study demonstrating that climate adaptation is genetically complex, the authors identify over 40 climate-associated genes and infer their biological roles. Similar approaches could help predict the ability of populations to adapt and thus assess their vulnerability to climate change (Bay et al., 2018).

Predicting evolutionary responses to climate change requires predicting patterns of evolutionary potential and selection under new or novel conditions. How genetic variance fluctuates across environments (G × E), populations, and traits is still poorly understood (Saltz et al., 2018). Because selection and gene flow among populations can shape evolutionary potential, space-for-time substitution experiments can offer some insights into the expected changes in additive genetic variance. For example, in wild birds, the evolutionary potential for morphological traits has been suggested to be highest when local habitat conditions are close to the niche optimum but not too close (intermediate habitat favorability, Martínez‐Padilla et al., 2017). Similarly, Chantepie et al. (2024, 29–42) show that in great tits, the genetic (co)variances of life history (e.g., phenology, reproduction, etc.), but not morphological traits, are shaped by the climatic conditions. This directly supports the conclusions from the genomic study by Stonehouse et al. (2024, 18–28). However, Nadeau and Urban (2024, 43–55) present a cautionary tale regarding predicting evolutionary potential based on the selection history of wild populations of *Daphnia magna*. Despite clear expectations of how spatial and temporal variation in temperature should shape genetic variation of fitness and critical thermal maximum, no such pattern was detected. Our predictions of evolutionary potential are still often based on verbal models because numeric predictions are extremely complex to devise. Basing predictions of evolutionary potential on empirical estimations of selection and gene flow (Arnold et al., 2008; Chantepie & Chevin, 2020) would be a challenging but necessary next step.

Local and global climates are known to be major drivers of selection pressures (Siepielski et al., 2017). Consequently, climate change is expected to alter selection pressures, including intensifying ongoing ones, such as stronger selection for earlier breeding under extreme climatic events (Marrot et al., 2017) or altering them radically, for example leading to winter diapause counter selection (Tougeron et al., 2020). Other environmental features may also strongly affect selection patterns. In support of this, Sauve et al. (2024, 56–63) show the intensity of selection on growth in black-legged kittiwakes (*Rissa tridactyla*) fluctuates according to air temperature. Thanks to a long-term feeding experiment in a wild population, they also demonstrate how variable resource dynamics can alter and even locally buffer selection. The importance of environmental conditions is further supported in a study by Nomoto et al. (2024, 114–127) investigating the effects of competition within plant communities. They utilize a transplant experiment along an altitudinal gradient and estimate shifts in directional selection on alpine plant morphology and phenology in response to climate and competition. Their results highlight that by depressing fitness, competitive interactions may limit the potential for selection. This study thus demonstrates that future studies should aim to also understand the more indirect effects of climate change, such as changing biotic interactions, on the potential for evolutionary rescue of natural populations. Doing so will be key to teasing apart the contribution of different sources of environmental heterogeneity in shaping selection and ultimately evolutionary responses.

An equally important aspect of predicting evolutionary responses to climate change requires improving our understanding of the evolution of plasticity itself since environmental cues can be altered by climate change (Bonamour et al., 2019), and the expression of plasticity in extreme situations might reach its limits. These questions are often discussed in the context of plasticity in continuous traits (e.g., Chevin & Hoffmann, 2017), but less discussed for discrete traits that can be of major importance (e.g., environment-dependent sex determination, color morph, etc.) (Reid & Acker, 2022). Crowther et al. (2024, 64–75) investigate how plasticity in discrete traits impacts evolutionary responses to environmental change. Environmental sex determination is common in many taxa (e.g., reptiles and teleost fishes) where the temperature experienced during embryonic or larval development determines the sex of the offspring. Interestingly, sex determination can be plastic in different ways (visualized via the slope of a nonlinear latent sex ratio reaction norm and a linear reaction norm of the temperature threshold producing either sex). The authors demonstrate how both types of plasticity differently affect the evolution of the threshold in response to climate change. For example, while a steep latent plasticity promotes the evolution of the threshold, linear plasticity of this threshold actually hampers its evolution. Hence, the type of ancestral plasticity will be crucial in determining the role of plasticity in facilitating or hindering evolution.

The role of plasticity in constraining or promoting evolved responses following environmental change is important for understanding how traits in general are going to adapt to climate change (Ghalambor et al., 2007; Noble et al., 2019). In an elegant experiment, Swaegers et al. (2024, 76–88) show that populations of the damselfly (*Ischnura elegans*) in a southward expansion from France into Spain have evolved increased heat tolerance compared with French core populations of the same species. By manipulating heat tolerance in the southward expanding populations via the use of a hypermethylating agent, the authors are able to significantly increase their heat tolerance beyond those typically measured in an older Spanish expansion zone. Thus, recent migrants are more plastic relative to older migrants. Experiments such as those conducted by Swaegers et al. (2024, 76–88) demonstrate that epigenetic and therefore plastic responses can be critical during the early stages of range shifts, but that genetic adaptations likely prevail over time.

## Harnessing the power of evolutionary history

The notion of using evolutionary history to inform future predictions is based on the premise that history often repeats itself. Evolutionary history can influence our predictions in various ways. The previous section emphasized how selection pressures may shape evolutionary potential, but more complex scenarios can arise. In addition to Swaegers et al. (2024, 76–88), two other studies from this Special Issue emphasize how species and population-specific history can affect responses to ongoing climate change, either through changes in distribution range or in situ adaptive responses. Predicting whether species will shift their distribution range or adapt locally is an important question as the requirements and limitations of each are different (e.g., need for habitat corridors vs. genetic variance). Moreover, the consequences of range shifts are evolutionarily important because they may affect the evolutionary trajectory of entire systems when the shifting species encounters novel habitats and novel competitors/communities (Suárez et al., 2022). In order to generate better predictions of how such evolutionary trajectories may unfold in response to climate change, we need candidate predictors that influence the extent and probability of range shifts. Hällfors et al. (2024, 89–100) ask whether adaptation to climate niche in the past, a proxy for tolerance to changing environmental conditions, can predict a poleward range shift of 283 species of moths, butterflies and birds in Finland. Based on nationwide long-term monitoring data over two decades, they find that birds and moths with narrower climatic niches display stronger northward shifts. Surprisingly, they find an opposite pattern in butterflies in relation to moisture niche. This finding is critical because such large-scale patterns make it possible to detect general trends and also provide insight into potential proximate causes (adaptation to thermal and moisture regimes) driving climate change adaptation.

Another way through which evolutionary history can inform future predictions is through studies of sexual selection. In particular, the role of sexual selection in evolutionary rescue has been subject to strong debate wherein sexual selection can reduce the effective population size and lead to sexual conflicts impeding natural selection. Sexual selection may also accelerate adaptation to novel environments by increasing the breeding success of better-adapted individuals (e.g., Arnqvist & Rowe, 2005). Fueled by these ideas, several reviews have synthesized the effect of the interplay between natural and sexual selection on the rate of adaptation, especially in the context of changing temperatures (e.g., Candolin & Heuschele, 2008; Pilakouta & Ålund, 2021). Baur et al. (2024, 101–113) offer a fresh perspective by asking how the history of sexual selection affects thermal sensitivity, since the expression of sexually selected traits may reduce stress tolerance. Using long-term experimental evolution, the authors assess how different levels of sexual and natural selection affect male fertility under acute heat stress in the seed beetle (*Callosobruchus maculatus*). The performance of males with a history of polyandry is most affected by stressful thermal conditions as are their female counterparts. In particular, the experiment demonstrated that heat stress and sexual selection together may exacerbate species vulnerability to climate change. These results highlight the need to integrate the effects of sexual selection not only as an ongoing process but also in terms of how it can affect organismal trade-offs, particularly between postcopulatory traits (e.g., sperm competition) and fertility.

## No organism is an island . . . The importance of interactions between mating partners and beyond

Interactions between organisms have a strong potential to affect responses to climate change. For example, competition may affect the intensity of selective pressures (Nomoto et al., 2024, 114–127; Sauve et al. (2024, 56–63) or shape adaptive potential (Baur et al., 2024, 101–113). Kulmuni et al. (2024, 128–136) suggest that hybrid mating interactions among closely related species will be of importance as well. Because the generation of new adaptive mutations is a slow process and standing genetic variation may not be sufficient for small or isolated populations, hybridization can “fast track” the generation of adaptive genetic variance. Even though hybrids are generally associated with having lower fitness, which can play an important role in maintaining barriers between species, there are accumulating empirical reports highlighting the adaptive potential of hybridization (e.g., Martin-Roy et al., 2021). Kulmuni et al. (2024, 128–136) propose that strongly changing environments may increase the occurrence of hybrid vigor. Using both modeling and simulations, they show that hybrids of both haploid and diploid populations adapt faster to a rapidly changing environment relative to parental populations in virtually all models. As reflected here, current studies involve mostly dyadic interactions, such as mating partners, but there is a significant need to integrate a network of social interactions that include, for example, helpers or competitors (from the same or a different species).

Recently, much emphasis has been placed on understanding how social relationships can affect evolutionary trajectories (and ultimately population persistence) both through selection (e.g., Fisher et al., 2017) and their effects on evolutionary potential (e.g., Baud et al., 2022). In this Special Issue, two contributions evaluate the role of interactions between mating partners in population persistence. Focusing on laying date, a textbook example of adaptive phenological response to climate change, Murray et al. (2024, 137–148) investigate whether the male partner can affect the rate of evolutionary response of laying date and hence the maximum sustainable rate of environmental change (cf/sensu Chevin et al., 2010). If females are expressing a plastic response to male phenotype, then the evolution of the male phenotype can have a strong impact on population persistence, facilitating or hindering population adaptation depending on the genetic correlations between sexes. Gómez-Llano et al. (2024, 149–160) ask whether male harm could influence adaptation and evolutionary rescue to a changing environment. Using a meta-analysis approach, the authors demonstrate that male harm in general exerts negative effects on female fitness, the extent of which depends on the type of harm (e.g., male harassment vs. traumatic insemination). The authors develop a theoretical model around these findings and further find that population decline can be reduced when sexual conflict influences local adaptation, but at the expense of genetic adaptation. The authors describe this trade-off as a double-edged sword where male harm can buffer demographic costs (e.g., less adapted males exert weaker harm on females) of climate change, but in turn delay genetic adaptation and consequently evolutionary rescue. An interesting twist to this story, however, is that variation in mating systems and the kind of harm that males exert on females can mitigate this trade-off, suggesting that eco-evolutionary processes that promote such variation will be critical to facilitate evolutionary rescue in systems where male harm is prominent. Consequently, both Gómez-Llano et al. (2024, 149–160) and Baur et al. (2024, 101–113) highlight the complex effects of social interactions on evolutionary trajectories.

## Conclusion

In general, predictions are still hampered by a lack of integration among population dynamics, individual responses, and evolutionary responses (Johnston et al., 2019). This is highlighted by the contribution of Crino et al. (2024, 161–171) presenting a conceptual framework centered on glucocorticoids, a major stress hormone in vertebrates mediating, among other things, responses to thermal stress. Physiological and behavioral responses to glucocorticoids have short-term adaptive effects, but their effects on fitness become more complex under chronic stress. The longer-term effects can also include both adaptive and maladaptive transgenerational consequences (Crino et al., 2024, 161–171). Understanding the interplay between the pleiotropic effects of glucocorticoids as well as between the different time scales will provide keys to understand fitness variations and predict selection patterns as well as evolutionary trajectories.

The diversity of evolutionary responses to climate change documented in this Special Issue clearly demonstrates that the solution to understanding “when” and “how” we can predict adaptive responses is as complex as the scope of the problem. To gain a perspective of the problem and its solutions, the concluding paper by Urban et al. (2024, 172–187) builds on the views of many contributors to this Special Issue to highlight key challenges to advancing research on factors that promote evolutionary rescue (e.g., the capacity of systems to adapt to a rapidly changing environment). In particular, we need to investigate multiple traits simultaneously to gain insights into the potential changes in trait-space, to better understand when plasticity reaches its limits or hinders adaptive evolution, including plasticity evolution (Iler et al., 2013).

Urban et al. (2024, 172–187) also highlight the diversity of methods needed to address questions related to climate change adaptation. This is also evident in the diversity of methods used in the contributions of the Special Issue in general. In the future, experimental evolution, resurrection, and transplant experiments will play an enormously important role in unpacking adaptive capacity and the molecular bases of adaptive responses, particularly in conjunction with novel genomic tools. Similarly, long-term monitoring programs of individuals or communities will be critical to shed light on how they are responding to climate change in general and how selection promotes evolutionary rescue. Using data from long-term monitoring programs to test against new data will play a key role in forecasting long-term evolutionary change.

Merging the challenges mentioned above with the perspectives of many of the contributors to this Special Issue, Urban et al. (2024, 172–187) outline a road map for future research by providing key actions that will enable predictions of evolutionary change in response to climate change. We hope that outlining these actions will lead to important research that seeks to fill important gaps that currently hamper our ability to inform future predictions. We therefore anticipate these actions will ignite investigations of how the effects of climate change unfold from phenotypes to genotypes and the selective forces that produce evolutionary rescue in general.

## Data Availability

There is no data to be archived.

## References

[CIT0001] Arnold, S. J. J., Bürger, R., Hohenlohe, P. A., Ajie, B. C., & Jones, A. G. (2008). Understanding the evolution and stability of the G-matrix. Evolution, 62, 2451–2461. 18973631 10.1111/j.1558-5646.2008.00472.x PMC3229175

[CIT0002] Arnqvist, G., & Rowe, L. (2005). Sexual conflict. Princeton University Press.

[CIT0003] Arrhenius, S. (1896). On the influence of carbonic acid in the air upon the temperature of the ground. The London, Edinburgh, and Dublin Philosophical Magazine and Journal of Science, 41(251), 237–276. 10.1080/14786449608620846

[CIT0004] Baud, A., McPeek, S., Chen, N., & Hughes, K. A. (2022). Indirect genetic effects: A cross-disciplinary perspective on empirical studies. The Journal of Heredity, 113(1), 1–15. 10.1093/jhered/esab059 34643239 PMC8851665

[CIT0005] Baur, J., Zwoinska, M., Koppik, M., Snook, R. R., & Berger, D. (2024). Heat stress reveals a fertility debt owing to postcopulatory sexual selection. Evolution Letters, 8(1), 101–113. 10.1093/evlett/qrad007. Advance access publication 16 March 2023. PMC10872150 38370539

[CIT0006] Bay, R. A., Harrigan, R. J., Underwood, V. L., Gibbs, H. L., Smith, T. B., & Ruegg, K. (2018). Genomic signals of selection predict climate-driven population declines in a migratory bird. Science, 359(6371), 83–86. 10.1126/science.aan4380 29302012

[CIT0007] Bonamour, S., Chevin, L., Charmantier, A., & Teplitsky, C. (2019). Phenotypic plasticity in response to climate change: The importance of cue variation. Philosophical Transactions of the Royal Society B: Biological Sciences, 374(1768), 1768. 10.1098/rstb.2018.0178 PMC6365871 30966957

[CIT0008] Bonnet, T., Morrissey, M. B., Morris, A., Morris, S., Clutton-Brock, T. H., Pemberton, J. M., & Kruuk, L. E. B. (2019). The role of selection and evolution in changing parturition date in a red deer population. PLoS Biology, 17(11), e3000493. 10.1371/journal.pbio.3000493 31689300 PMC6830748

[CIT0009] Bradshaw, W. E., & Holzapfel, C. M. (2001). Genetic shift in photoperiodic response correlated with global warming. Proceedings of the National Academy of Sciences of the United States of America, 98(25), 14509–14511. 10.1073/pnas.241391498 11698659 PMC64712

[CIT0010] Candolin, U., & Heuschele, J. (2008). Is sexual selection beneficial during adaptation to environmental change? Trends in Ecology and Evolution, 23(8), 446–452. 10.1016/j.tree.2008.04.008 18582989

[CIT0011] Chantepie, S., Charmantier, A., Delahaie, B., Adriaensen, F., Matthysen, E., Visser, M. E., Álvarz, E., Barba, E., Orell, M., Sheldon, B., Ivankina, E., Kerimov, A., Lavergne, S., & Teplitsky, C. (2024). Divergence in evolutionary potential of life-history traits among wild populations is predicted by differences in climatic conditions. Evolution Letters, 8(1), 29–42. 10.1093/evlett/qrad067 PMC10872211 38370542

[CIT0012] Chantepie, S., & Chevin, L. M. (2020). How does the strength of selection influence genetic correlations? Evolution Letters, 4(6), 468–478. 10.1002/evl3.201 33312683 PMC7719553

[CIT0013] Chevin, L., & Hoffmann, A. A. (2017). Evolution of phenotypic plasticity in extreme environments. Philosophical Transactions of the Royal Society of London, Series B: Biological Sciences, 372(1723), 20160138. 10.1098/rstb.2016.0138 28483868 PMC5434089

[CIT0014] Chevin, L., Lande, R., & Mace, G. M. (2010). Adaptation, plasticity, and extinction in a changing environment: Towards a predictive theory. PLoS Biology, 8(4), e1000357. 10.1371/journal.pbio.1000357 20463950 PMC2864732

[CIT0015] Crino, O. L., Bonduriansky, R., Martin, L. B., & Noble, D. W. A. (2024). A conceptual framework for understanding stress-induced physiological and transgenerational effects on population responses to climate change. Evolution Letters, 8(1), 161–171. 10.1093/evlett/qrad037. Advance access publication 29 September 2023. PMC10871929 38370553

[CIT0016] Crowther, C., Stephen, B., & Schwanz, L. (2024). Plasticity and the adaptive evolution of switchlike reaction norms under environmental change. Evolution Letters, 8(1), 64–75. 10.1093/evlett/qrad035. Advance access publication 31 August 2023. PMC10872057 38370546

[CIT0017] Díaz, S., Settele, J., Brondízio, E. S., Ngo, H. T., Agar, J., Arneth, A., Balvanera, P., Brauman, K. A., Butchart, S. H. M., Chan, K. M. A., et al. (2019). Pervasive human-driven decline of life on Earth points to the need for transformative change. Science, 366, eaax3100. 10.1126/science.eaax3100 31831642

[CIT0018] Fisher, D. N., Boutin, S., Dantzer, B., Humphries, M. M., Lane, J. E., & McAdam, A. G. (2017). Multilevel and sex-specific selection on competitive traits in North American red squirrels. Evolution, 71(7), 1841–1854. 10.1111/evo.13270 28543051

[CIT0019] Fraixedas, S., Roslin, T., Antão, L. H., & Laine, A. -L. (2022). Nationally reported metrics can’t adequately guide transformative change in biodiversity policy. Proceedings of the National Academy of Sciences of the United States of America, 119, e2117299119. 35217615 10.1073/pnas.2117299119 PMC8892539

[CIT0020] Ghalambor, C. K., McKay, J. K., Carroll, S. P., & Reznick, D. N. (2007). Adaptive vs non-adaptive phenotypic plasticity and the potential for contemporary adaptation in new environments. Functional Ecology, 21(3), 394–407. 10.1111/j.1365-2435.2007.01283.x

[CIT0021] Gienapp, P., Postma, E., & Visser, M. E. (2006). Why breeding time has not responded to selection for earlier breeding in a songbird population. Evolution, 60(11), 2381–2388. 17236428

[CIT0022] Gómez-Llano, M., Faria, G. S., García-Roa, R., Noble, D. W. A., & Carazo, P. (2024). Male harm suppresses female fitness, affecting the dynamics of adaptation and evolutionary rescue. Evolution Letters, 8(1), 149–160. 10.1093/evlett/qrac002. Advance access 31 January 2023. PMC10871930 38370549

[CIT0023] Hällfors, M., Heikkinen, R. K., Kuussaari, M., Lehikoinen, A., Luoto, M., Pöyry, J., Virkkala, R., Saastamoinen, M., & Kujala, H. (2024). Recent rangeshifts of moths, butterflies, and birds are driven by the breadth of their climate niche. Evolution Letters, 8(1), 89–100. 10.1093/evlett/qrad004. Advance access publication 12 March 2023. PMC10872046 38370541

[CIT0024] Iler, A. M., Høye, T. T., Inouye, D. W., & Schmidt, N. M. (2013). Nonlinear flowering responses to climate: Are species approaching their limits of phenological change? Philosophical Transactions of the Royal Society of London, Series B: Biological Sciences, 368(1624), 20120489. 10.1098/rstb.2012.0489 23836793 PMC3720060

[CIT0025] Inouye, D. W. (2022). Climate change and phenology. Wiley Interdisciplinary Reviews: Climate Change, 13(3), 1–17.

[CIT0026] IPBES. (2019). Global assessment report on biodiversity and ecosystem services of the Intergovernmental Science-Policy Platform on Biodiversity and Ecosystem Services (p. 1148). In E. S. Brondizio, J. Settele, S. Díaz, & H. T. Ngo (Eds.), IPBES Secretariat. 10.5281/zenodo.3831673

[CIT0027] IPCC. (2022). Climate change 2022: Impacts, adaptation, and vulnerability. UNEP.

[CIT0028] Johnston, A. S. A., Boyd, R. J., Watson, J. W., Paul, A., Evans, L. C., Gardner, E. L., & Boult, V. L. (2019). Predicting population responses to environmental change from individual-level mechanisms: Towards a standardized mechanistic approach. Proceedings of the Royal Society B: Biological Sciences, 286(1913), 20191916. 10.1098/rspb.2019.1916 PMC6834044 31615360

[CIT0029] Kruuk, L. E. B., Slate, J., & Wilson, A. J. (2008). New answers for old questions: The evolutionary quantitative genetics of wild animal populations. Annual Review of Ecology, Evolution, and Systematics, 39, 525–548.

[CIT0030] Kulmuni, J., Wiley, B., & Otto, S. (2024). On the fast track: Hybrids adapt more rapidly than parental populations in a novel environment. Evolution Letters, 8(1), 128–136. 10.1093/evlett/qrad002. Advance access publication 1 March 2023. PMC10871894 38370548

[CIT0031] Marquet, P. A., Naeem, S., Jackson, J. B. C., & Hodges, K. (2019). Navigating transformation of biodiversity and climate. Science Advances, 5(11), a0969. 10.1126/sciadv.aba0969 PMC6891924 31832538

[CIT0032] Marrot, P., Garant, D., & Charmantier, A. (2017). Multiple extreme climatic events strengthen selection for earlier breeding in a wild passerine. Philosophical Transactions of the Royal Society of London, Series B: Biological Sciences, 372(1723), 20160372. 10.1098/rstb.2016.0372 28483864 PMC5434099

[CIT0033] Martínez‐Padilla, J., Estrada, A., Early, R., & Garcia-Gonzalez, F. (2017). Evolvability meets biogeography: Evolutionary potential decreases at high and low environmental favourability. Proceedings of the Royal Society B: Biological Sciences, 284, 20170516. 10.1098/rspb.2017.0516 PMC5474074 28615500

[CIT0034] Martin-Roy, R., Nygård, E., Nouhaud, P., & Kulmuni, J. (2021). Differences in thermal tolerance between parental species could fuel thermal adaptation in hybrid wood ants. American Naturalist, 198(2), 278–294. 10.1086/715012 34260873

[CIT0035] Merilä, J., & Hendry, A. P. (2014). Climate change, adaptation, and phenotypic plasticity: The problem and the evidence. Evolutionary Applications, 7(1), 1–14. 10.1111/eva.12137 24454544 PMC3894893

[CIT0036] Moiron, M., Teplitsky, C., Haest, B., Charmantier, A., & Bouwhuis, S. (2024). Micro-evolutionary response of spring migration timing in a wild seabird. Evolution Letters, 8(1), 8–17. 10.1093/evlett/qrad014. Advance access publication 3 May 2023. PMC10872114 38370547

[CIT0037] Murray, M., Wright, J., & Araya-Ajoy, Y. G. (2024). Evolutionary rescue from climate change: Male indirect genetic effects on lay-dates and their consequences for population persistence. Evolution Letters, 8(1), 137–148. 10.1093/evlett/qrad022. Advance access publication 13 July 2023. PMC10939382 38487362

[CIT0038] Nadeau, C. P., & Urban, M. C. (2024). Macroecological predictors of evolutionary and plastic potential do not apply at microgeographic scales for a freshwater *cladoceran* under climate change. Evolution Letters, 8(1), 43–55. 10.1093/evlett/qrad042. Advance access publication 12 October 2023. PMC10872021 38370540

[CIT0039] Nadeau, C. P., Urban, M. C., & Bridle, J. R. (2017). Climates past, present, and yet-to-come shape climate change vulnerabilities. Trends in Ecology and Evolution, 32(10), 786–800. 10.1016/j.tree.2017.07.012 28844791

[CIT0040] Noble, N. W. A., Radersma, R., & Uller, T. (2019). Plastic responses to novel environments are biased towards phenotype dimensions with high additive genetic variation. Proceedings of the National Academy of Sciences of the United States of America, 116, 13452–13461. 31217289 10.1073/pnas.1821066116 PMC6613099

[CIT0041] Nomoto, H., Simone, F., & Alexander, J. (2024). Competitors alter selection on alpine plants exposed to experimental climate change. Evolution Letters, 8(1), 114–127. 10.1093/evlett/qrad066 PMC10871967 38370552

[CIT0042] Pelletier, F., Garant, D., & Hendry, A. P. (2009). Eco-evolutionary dynamics. Philosophical Transactions of the Royal Society of London, Series B: Biological Sciences, 364(1523), 1483–1489. 10.1098/rstb.2009.0027 19414463 PMC2690510

[CIT0043] Pilakouta, N., & Ålund, M. (2021). Sexual selection and environmental change: What do we know and what comes next? Current Zoology, 67(3), 293–298. 10.1093/cz/zoab021 34616921 PMC8488989

[CIT0044] Pujol, B., Blanchet, S., Charmantier, A., Danchin, E., Facon, B., Marrott, P., Roux, F., Scotti, I., Teplitsky, C., Thomson, C. E., & Winney, I. (2018). The missing response to selection in the wild. Trends in Ecology and Evolution, 33, 337–346. 29628266 10.1016/j.tree.2018.02.007 PMC5937857

[CIT0045] Ramakers, J. J. C., Gienapp, P., & Visser, M. E. (2019). Phenological mismatch drives selection on elevation, but not on slope, of breeding time plasticity in a wild songbird. Evolution, 73, 175–187.

[CIT0046] Reid, J. M., & Acker, P. (2022). Properties of phenotypic plasticity in discrete threshold traits. Evolution, 76(2), 190–206. 10.1111/evo.14408 34874068

[CIT0047] Roff, D. A. (2007). Centennial celebration for quantitative genetics. Evolution, 61, 1017–1032. 17492957 10.1111/j.1558-5646.2007.00100.x

[CIT0048] Saltz, J. B., Bell, A. M., Flint, J., Gomulkiewicz, R., Hughes, K. A., & Keagy, J. (2018). Why does the magnitude of genotype-by-environment interaction vary? Ecology and Evolution, 8(12), 6342–6353. 10.1002/ece3.4128 29988442 PMC6024136

[CIT0057] Sauve, D., A. Charmantier, A., Hatch, S. A. and Friesen, V. L. (2024). The magnitude of selection of growth varies among years and increases under warming conditions in a subarctic seabird. *Evolution Letters*, 8(1), 56–63. 10.1093/evlett/qrad001 PMC10871900 38370550

[CIT0049] Scheffers, B. R., De Meester, L., Bridge, T. C. L., Hoffmann, A. A., Pandolfi, J. M., Corlett, R. T., Butchart, S. H. M., Pearce-Kelly, P., Kovacs, K. M., Dudgeon, D., et al. (2016). The broad footprint of climate change from genes to biomes to people. Science, 354, aaf7671. 10.1126/science.aaf7671 27846577

[CIT0050] Siepielski, A. M., Morrissey, M. B., Buoro, M., Carlson, S. M., Caruso, C. M., Clegg, S. M., Coulson, T., DiBattista, J., Gotanda, K. M., Francis, C. D., Hereford, J., Kingsolver, J. G., Augustine, K. E., Kruuk, L. E. B., Martin, R. A., Sheldon, B. C., Sletvold, N., Svensson, E. I., Wade, M. J., & MacColl, A. D. C. (2017). Precipitation drives global variation in natural selection. Science, 355(6328), 959–962. 10.1126/science.aag2773 28254943

[CIT0051] Stonehouse, J. C., Spurgin, L. G., Laine, V. N., Bosse, M., Groenen, M. A. M., van Oers, K., Sheldon, B. C., & Visser, M. E. (2024). The genomics of adaptation to climate in European great tit (*Parus major*) populations. Evolution Letters, 8(1), 18–28. 10.1093/evlett/qrad043. Advance access publication 12 October 2023. PMC10872194 38370545

[CIT0052] Suárez, D., Arribas, P., Jiménez-García, E., & Emerson, B. C. (2022). Dispersal ability and its consequences for population genetic differentiation and diversification. Proceedings of the Royal Society B: Biological Sciences, 289, 20220489. 10.1098/rspb.2022.0489 PMC9115014 35582805

[CIT0053] Swaegers, J., De Cupere, S., Gaens, N., Lancaster, L. T., Carbonell, J. A., Sánchez Guillén, R. A., & Stokes, R. (2024). Plasticity and associated epigenetic mechanisms play a role in thermal evolution during range expansion. Evolution Letters, 8(1), 76–88. 10.1093/evlett/qrac007. Advance access publication 31 January 2023. PMC10872138 38370551

[CIT0054] Tougeron, K., Brodeur, J., Le Lann, C., & Baaren, J. van. (2020). How climate change affects the seasonal ecology of insect parasitoids. Ecological Entomology, 45, 167–181.

[CIT0055] Urban, M. C., Swaegers, J., Stokes, R., Snook, R. R., Otto, S. P., Noble, D. W. A., Moiron, M., Hällfors, M. H., Gómez-Llano, M., Fior, S., Cote, J., Charmantier, A., Bestion, E., Berger, D., Baur, J., Alexander, J. M., Saastamoinen, M., Edelsparre, A. H., & Teplitsky, C. (2024). When and how can we predict adaptive responses to climate change. Evolution Letters, 8(1), 172–187. 10.1093/evlett/qrad038. Advance access publication 29 November 2023. PMC10872164 38370544

[CIT0056] Walther, G.-R., Post, E., Convey, P., Menzel, A., Parmesan, C., Beebee, T. J. C., Fromentin, J. -M., Hoegh-Guldberg, O., & Bairlein, F. (2002). Ecological responses to recent climate change. Nature, 416(6879), 389–395. 10.1038/416389a 11919621

